# MicroRNAs in sensorineural diseases of the ear

**DOI:** 10.3389/fnmol.2013.00052

**Published:** 2013-12-23

**Authors:** Kathy Ushakov, Anya Rudnicki, Karen B. Avraham

**Affiliations:** Department of Human Molecular Genetics and Biochemistry, Sackler Faculty of Medicine and Sagol School of Neuroscience, Tel Aviv UniversityTel Aviv, Israel

**Keywords:** deafness, inner ear, cochlea, vestibule, microRNAs

## Abstract

Non-coding microRNAs (miRNAs) have a fundamental role in gene regulation and expression in almost every multicellular organism. Only discovered in the last decade, miRNAs are already known to play a leading role in many aspects of disease. In the vertebrate inner ear, miRNAs are essential for controlling development and survival of hair cells. Moreover, dysregulation of miRNAs has been implicated in sensorineural hearing impairment, as well as in other ear diseases such as cholesteatomas, vestibular schwannomas, and otitis media. Due to the inaccessibility of the ear in humans, animal models have provided the optimal tools to study miRNA expression and function, in particular mice and zebrafish. A major focus of current research has been to discover the targets of the miRNAs expressed in the inner ear, in order to determine the regulatory pathways of the auditory and vestibular systems. The potential for miRNAs manipulation in development of therapeutic tools for hearing impairment is as yet unexplored, paving the way for future work in the field.

## INTRODUCTION

Hearing loss (HL) is the most prominent neurosensory disorder in humans. Congenital deafness affects at least one in 500 newborns and more than half of these cases are hereditary (National Institutes of Health, NIDCD)^1^. As HL is also age dependent, more individuals can be affected at later stages of their lives. The ear is a complex transducing organ, which consists of both exterior and interior parts. Vibrations of the middle ear’s bones mirroring incoming sounds are translated into vibration of the basilar membrane, which in turn leads to mechanotransduction at the organ of Corti in specified cells, the hair cells. Mammalian auditory hair cells, surrounded by non-sensory supporting cells, are the main functional components of the cochlea. They are organized in three rows of outer hair cells (OHC) and one row of inner hair cells (IHC). Their apical actin-based microvilli are referred to as stereocilia. The mechanical stimulus sensed by the stereocilia is converted into an action potential, which in turn transfers the detected sound to the brain (Kelley, 2006). Specifically, coding of sound travels to the higher auditory systems via the brainstem, where there are synapses in the cochlear nuclei and the superior olivary complex (SOC), to the inferior colliculus of the midbrain and finally to the auditory cortex.

For many years, the conventional dogma in molecular biology defined the mammalian genome as one containing protein-coding genes and other repetitive and non-transcribed sequences. The latter was deemed to be non-essential, unless directly involved in RNA synthesis. The last decade has completely reversed this view and the field of non-coding RNAs (ncRNAs) has undergone a dramatic metamorphosis as a portion of these RNAs, microRNAs (miRNAs) are now recognized as having a vital role in gene expression and function. The first recognized miRNAs were lin-7 and let-7 in *Caenorhabditis elegans* (Lagos-Quintana et al., 2001), but since then the number of these regulatory RNAs has grown to 30,424 mature miRNA sequences in 206 species (Kozomara and Griffiths-Jones, 2011)^2^. miRNAs are the most studied and understood forms of ncRNAs, and have been shown to fulfill regulatory functions in many species, including the mammalian system.

miRNAs are small ~23 nucleotide long RNA species. Pri-miRNAs are transcribed together with other forms of RNA by RNA polymerase II and processed through the Drosha–Dicer pathway (Carthew and Sontheimer, 2009). While still in the nucleus, pri-miRNAs are cleaved by Drosha and exported to the cytoplasm via exportin 5. The product of the cleavage pre-miRNA hairpin is composed of the main -5p and the complementary -3p (formally star) strands that are connected by the stem loop. In the cytoplasm, the pre-miRNA is cleaved by a second enzyme, Dicer, to produce the mature miRNA. miRNAs possess a seed region of 7 nt that determines its target specificity (Bartel, 2009). Upon sequence complementarity, this region will bind to sequences at the 3′ untranslated region (UTR) of target genes. In this fashion, miRNAs inhibit target mRNAs by translational repression and mRNA destabilization (Guo et al., 2010) and regulate gene expression through the RNA interference (RNAi) pathway. Another group of ncRNAs, long intervening noncoding RNAs (lincRNAs), while more elusive in their classification, are considered to have expansive roles in gene regulation (Ulitsky and Bartel, 2013).

How have ncRNAs contributed to the study of the auditory and vestibular systems? miRNAs were first described in the zebrafish inner ear in 2005 (Wienholds et al., 2005), which heralded a number of studies in the mammalian inner ear worldwide. The study of lincRNAs has not yet advanced at the same pace.

## miRNAs IN THE INNER EAR

**FIGURE 1:**
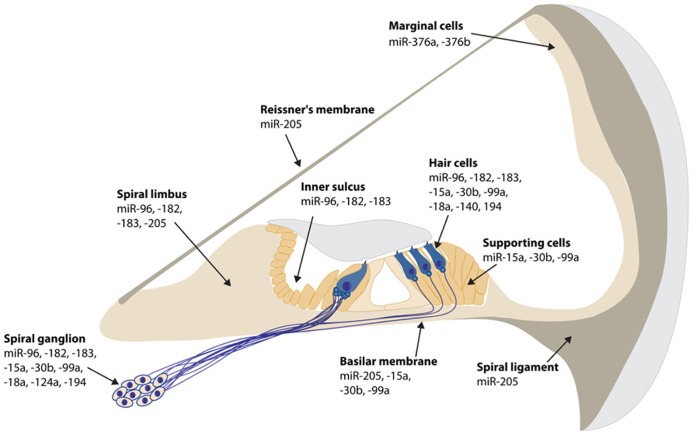
**Spatial expression patterns of miRNAs in the mouse cochlea.** The expression data is based on *in situ* hybridization experiments of P0 mouse inner ear sections, except for miR-194 at E16.5, miR-140 at P1 and miR-124a and -100a at P5. miR-200b is ubiquitously expressed in all epithelial cells in the inner ear, both in the cochlea and vestibule at P0 (Weston et al., 2006; Friedman et al., 2009; Sacheli et al., 2009; Soukup et al., 2009; Wang et al., 2010a,b; Elkan-Miller et al., 2011; Hertzano et al., 2011; Yan et al., 2012).

**FIGURE 2:**
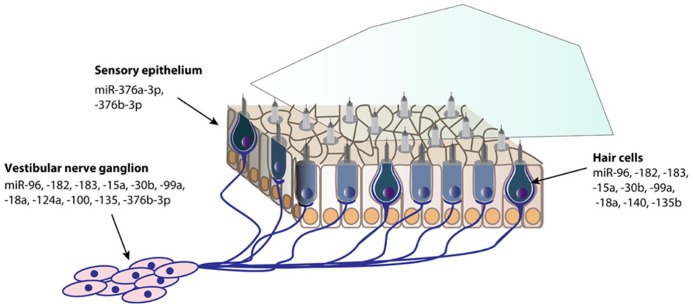
**Spatial expression patterns of miRNAs in the postnatal mouse vestibule.** The expression data is based on *in situ* hybridization experiments of P0 mouse inner ear sections, except for miR-140 at P1, and miR-124a and -100a at P5 (Weston et al., 2006; Friedman et al., 2009; Wang et al., 2010a; Elkan-Miller et al., 2011; Yan et al., 2012).

Since miRNAs have become an essential and fascinating aspect of gene regulation in the inner ear, hundreds of miRNAs have been identified using microarrays (Weston et al., 2006; Friedman et al., 2009; Wang et al., 2010a; Elkan-Miller et al., 2011; Zhang et al., 2013). The specific expression of a fraction of these miRNAs has been determined by *in situ* hybridization in the mouse inner ear (**Figures 1** and **2**). There are still many inner ear-expressing miRNAs waiting to be further characterized, both with regards to expression, targets and mechanisms.

The miR-183 family is the most characterized miRNA cluster in the inner ear. This conserved miRNA triad, composed of miR-183, miR-182, and miR-96, is transcribed in one polycistronic transcript. In both zebrafish and the mouse, the triad co-expressed in several neurosensory organs, including the ear, nose, and eye (Wienholds et al., 2005; Weston et al., 2006; Karali et al., 2007). A study demonstrating the role of the miR-183 family in zebrafish by reducing and increasing levels of miRNAs by morpholino (MO) or miRNA injection, respectively, revealed that the miR-183 cluster is crucial for inner ear hair cell and neuronal development (Li et al., 2010). While the miRNAs overlap in their function, given the similarity in their seed regions, they may have different targets, due to the differences in resulting phenotypes following overexpression of each. In the ENU diminuendo mouse with a miR-96 mutation (Lewis et al., 2009). the expression of all three miRNAs remained intact, indicating that the mutation did not disturb the biogenesis of the triad. The mutant mouse showed rapidly progressive HL and hair cell abnormalities. In a search for miR-96 targets, 12 were predicted by miRanda with stringent filtering and five were validated by luciferase assay analysis, Aqp5, Celsr2, Myrip, Odf2, and Ryk. Since the mutation changes miR-96 seed region, the study suggests that a new seed region was created, now binding to new targets, and therefore both loss of normal targets and gain of novel targets could be responsible for the phenotype. In a microarray comparing gene expression between the wild type and the mutant *diminuendo* mouse inner ears, 96 transcripts were significantly affected. Five genes were markedly down-regulated and strongly and specifically expressed in hair cells: Slc26a5 (prestin), Ocm (oncomodulin), Pitpnm1, Gfi1, and Ptprq. None of these genes has a miR-96 binding site, suggesting these are indirect downstream targets, and their change in expression may be causing the diminuendo phenotype.

Further studies on the diminuendo mouse found that miR-96 is responsible for the maturation of the stereocilia bundle of the inner and OHC (Kuhn et al., 2011). Moreover, the synaptic morphology of the mutant mice remained immature, suggesting that miR-96 is involved in cochlear auditory nerve formation.

Identification of targets is a key ingredient for deciphering the function of an miRNA. Several studies defined targets for members of the miR-183 triad. In a study on cells derived from mouse otocysts, miR-182 promoted differentiation of these cells to a hair cell-like fate (Wang et al., 2012). Moreover, the transcription factor Tbx1 was found to be a target of miR-182. Tbx1 is a critical gene in DiGeorge syndrome, with the phenotype of patients including ear and hearing abnormalities. Tbx1 mouse mutants exhibit severe inner ear defects. Therefore the tightly regulated transcriptional regulation of Tbx1 in the mammalian ear may be influenced in part by miR-182, providing a function in crucial inner ear developmental pathways.

Clic5, a chloride intracellular channel that is associated with stereocilia in the inner ear, was identified as a target of both miR-96 and miR-182 (Gu et al., 2013). Clic5-mutant mice stereocilia bear a resemblance to the morphology of the diminuendo ENU mouse described above, leading to an investigation of its connection to this triad. Clic5 contains a miR-96/182 binding site and its activity was confirmed by a luciferase assay. Liposome transfection of these miRNAs into auditory-cell derived HEI-OC1 led to a reduction of Clic5 at both mRNA and protein levels.

The triad clearly plays an important role in other sensory systems. Inactivation of the three miRNAs in the mouse led to multiple sensory defects, with an emphasis on the loss of this triad in the retina (Lumayag et al., 2013). Not only did the mice have progressive retinal degeneration and photoreceptor defects, but there were significant changes in overall retinal gene expression, as revealed by profiling of microarrays.

Another well-characterized and highly expressing miRNA in the brain, miR-124 (Lagos-Quintana et al., 2002), appears to have an essential role in the inner ear. miR-124 is expressed in the inner ear in neuronal cells in the spiral and vestibular ganglia (Weston et al., 2006). In a study on the differential expression of miRNAs between cochlear and vestibular sensory epithelia, miR-124 was one of the most highly differentially expressed miRNAs, with eightfold higher expression in the cochlea. This suggests a specific role and targets for miR-124 in the cochlear neurons of the inner ear (Elkan-Miller et al., 2011). A recent study, searching for miRNAs that are involved in age-related hearing loss (ARHL; see miRNAs in ARHL), compared differentially expressed miRNAs in sensory epithelia of two mouse strains, C57BL/6J and CBA/J, at several ages. miR-124 was one of four miRNAs that were significantly down regulated in both mouse strains at the age of 9 months, compared to postnatal day (P)21 (Zhang et al., 2013). While more information regarding the targets of miR-124 to elucidate its role in the inner ear is required, this miRNA should clearly have significant influence on gene regulation.

## LOSS OF *DICER* IN THE INNER EAR

Dicer is a ribonuclease RNase III-like enzyme that is localized in the nucleus and functions to process double-stranded RNA (dsRNA). Dicer products then exit to the cytoplasm and are further processed into mature miRNAs. Dicer ablation is lethal in zebrafish (Wienholds et al., 2005) and produces no viable embryos in mice (Bernstein et al., 2003).

Dicer has been exploited to study miRNA function in the inner ear. Several conditional knock out (CKO) models have been generated. The first ear-specific Dicer1 CKO was generated using Pax2::Cre for specific expression in regions where Pax2 is expressed (Soukup et al., 2009). Dicer1 was ablated in the inner ear, kidneys and midbrain, resulting in embryonic lethal mice. The CKO mice showed significant loss of most inner ear structures by embryonic day (E)17.5. Although there was initial and normal formation and growth of neurons, the nerves of the CKO animals were rapidly lost after the decay in miRNA expression in the afferent neurons.

The first viable mice were the Pou4f3::Cre-Dicer CKO mice, using Dicer1 to remove miRNAs from hair cells (Friedman et al., 2009). Pou4f3 was used to express Dicer1 specifically in these cells. The hair cells developed normally, but were degenerated by postnatal day 38. Those that survived at this stage had an aberrant morphology and were presumably dysfunctional. This was confirmed by auditory brainstem response (ABR) testing, which indicated that these mice were deaf. In addition, they showed moderate vestibular dysfunction. Scanning electron microscopy (SEM) demonstrated that the stereocilia of the auditory hair cells were either missing or fused.

Foxg1 was used for site-specific expression to generate Foxg1::Cre-Dicer CKO mice (Kersigo et al., 2011). Overall, these mice had a reduction in anterodorsal regions of the skull, leading to craniofacial abnormalities. As for the ear, it began to develop abnormally around E12.5, with a significant reduction in the size of the ear by E14.5 and in the size of the otocyst by E18.5. This change was concomitant with a reduction in the ossification of the ear and a smaller tympanic ring. miRNA-124 was found to be specifically reduced prior to loss of the neurosensory portions, suggesting this miRNA is required for normal neuronal development.

Another hair-cell specific CKO was generated using the gene responsible for hair cell agenesis, Atoh1, to create Atoh1::Cre-Dicer mice (Weston et al., 2011). Ablation of miRNAs in the hair cells resulted in a progressive loss of OHCs from the base to the apex, with OHCs being more prone to damage as compared to the IHCs. The gradient in the rigorousness of hair cell loss hints that there is also a gradient in the expression pattern of miRNAs along the cochlea.

To study the role of Dicer and subsequent loss of miRNAs in the central auditory pathway, Erg2::Cre-Dicer mice were created (Rosengauer et al., 2012). Work on this CKO demonstrated that Dicer is indispensable for the formation of the cochlear nucleus complex (CNC) and the SOC. In the same study, an additional CKO mouse, Atoh7::Cre-Dicer, was used to dissect later stages of CNC formation. The CNC was comparable to the wild-type mouse, suggesting that Dicer is not crucial for the formation of these structures during late embryonic stages.

It is important to note that when drawing conclusions about Dicer1 function at different stages of development, the tissue-specific ablation is gradual and is specific to the Cre promoter used. Therefore residual Dicer1 expression may exist, leading to a less severe phenotype than expected with removal of this essential enzyme. Furthermore, there is no specificity with respect to miRNAs and rather provide an “all or nothing effect.” For specific miRNAs, the approach taken to examine loss of miR-182 in the retina is a relevant approach (Jin et al., 2009), though not yet exploited in the inner ear.

## IDENTIFICATION OF miRNA-PROTEIN TARGET PAIRS

Identifying novel or known miRNAs that are involved in specific processes in the inner ear and in the auditory pathways is the relatively easy part of miRNA research. However, discerning the molecular mechanisms, or moreover, the direct targets, is considerably more tedious and challenging. This point is exemplified by the number of miRNAs that have been identified versus the number of validated miRNA targets in the inner ear (**Table 1**). Potential targets of any miRNA can be predicted via TargetScan (Lewis et al., 2005), MicroCosm (formally MirBase; Kozomara and Griffiths-Jones, 2011) and similar prediction programs. These algorithms find a match between the 7-nucleotide seed region of the miRNA and the 3′UTR target mRNAs. It is important to note that this method is based on bioinformatics and relies on sequence similarities between the miRNA and the mRNA. TargetScan and analogous programs cannot eliminate potential targets on the basis of tissue specificity.

**Table 1:** Validation of miRNA-gene targets found in the inner ear.

miRNA	Gene target	Experimental system used	Reference
miR-183	TAO kinase 1 (Taok1)	Rat cochlear organotypic cultures transfected with antisense morpholinos.	Patel et al. (2013)
	Early growth response 1 (Egr1)
	Insulin receptor substrate 1 (Irs1)
miR-182	SRY-box containing transcription factor (Sox2)	*In situ* hybridization; luciferase assay in HEK293 cells	Weston et al. (2011)
miR-182	T-box 1 (Tbx1)	Luciferase assay in COS1 cells; overexpression of miR in cultured otic progenitor/stem cells.	Wang et al. (2012)
miR-96, miR-182	Chloride intracellular channel 5 (Clic5)	Co-expression in mouse auditory HEI-OC1 cells; luciferase assay in A549 cells; down-regulation of target.	Gu et al. (2013)
miR-15a	Solute carrier family 12 (sodium/potassium/chloride transporters), member 2 (Slc12a2), Claudin (Cldn12) Brain-derived neurotrophic factor (Bdnf)	*In situ* hybridization; luciferase assay in HEK-293T cells.	Friedman et al. (2009)
miR-21	Phosphatase and tensin homolog (Pten)	Down-regulation of target in cholesteatoma; inhibition of miR.	Friedland et al. (2009), Cioffi et al. (2010)
miR-21	Programmed cell death 4 (Pdcd4)	Western blot on cholesteatoma skin samples.	Friedland et al. (2009)
miR-135b	PC4 and SFRS1 interacting protein 1 (Psip1-p75)	Luciferase assay and qRT-PCR on Cal51, breast carcinoma, cells; inhibition of miR.	Elkan-Miller et al. (2011)
miR-200b	Zinc finger E-Box binding homeobox 1 (Zeb1)	Global gene expression analysis; complementary patterns of expression validated with *in situ* and immunohistochemistry	Hertzano et al. (2011)

After the initial bioinformatic analyzes, each miRNA/gene target must be validated by experimental techniques. There are several approaches for this validation. The most commonly used *in vitro* technique is the luciferase assay. This quantitative assay system was developed originally to assess promoter strength. In the miRNA field this technique is used to study whether there is a direct interaction of a miRNA and a 3′UTR of a potential target gene. Typically the miRNA is cloned into one vector and a 3′UTR is cloned in-frame with luciferase. If the gene is a “true” target, there will be no bioluminescence. If the miRNA cannot interact with the 3′UTR, luciferase will be produced continuously. If a direct interaction between the miRNA and gene target is found, one must show that the mutation in the seed region of the miRNA can abolish the binding. To demonstrate that miR-182 is a direct target of Sox2, a luciferase assay was performed both with a luciferase reporter vector with the 3′UTR of Sox2 and a mutated version of the 3′UTR at the seed region of miR-182 (Weston et al., 2011). The mutated 3′UTR could not bind miR-182 and the decrease in luciferase activity that was observed in the wild-type construct was lost.

To demonstrate an interaction in a more “*in vivo*” approach, anti-miRNAs are used. These short oligonucleotides are used to transfect either cell lines or cochlear cultures, and quench the endogenous miRNAs. The outcome of the antagonism is then probed either at the mRNA level, using qRT-PCR, or at the protein level, using western blot analysis, of the gene target. After confirming direct binding between miR-182 and the Tbx1 3′UTR by luciferase assay, degradation of the target on an mRNA level was tested (Wang et al., 2012). Isolated IHC infected with rA-miR-182 and transfected miR-182 inhibitor were collected and harvested to explore Tbx1 transcription. In the presence of miR-182, the mRNA levels of Tbx1 were restored as compared to infected cells, suggesting target inhibition. Checking the expression level of the predicted target gene by western blot can also provide evidence for miRNA-gene target interaction. Skin samples from cholesteatoma patients and control individuals were analyzed for protein levels of the putative miR-21 targets, PTEN, and PCDC4 (Friedland et al., 2009). In 75% of the cases, there was a substantial reduction in the levels of both proteins, validating the predicted targets of the miRNA.

Target recognition may be compromised as a result of a mutation, as was suggested for some of the human miR-96 mutations (Mencia et al., 2009). Given a change in the nucleotides that define the specificity of the miRNA, the miRNA might lose its ability to regulate its original targets. This hypothesis was examined with the human miRNA mutations. As two different mutations in the seed region of MIR-96, it was appealing to consider whether there are any new acquired targets. However, they could not detect any targets that are regulated by the “new” seed region of either of the two mutations.

## MECHANISMS OF miRNA FUNCTION IN THE INNER EAR

Roles of miRNAs in the inner ear can be also studied through identification of the overall intracellular pathways they are involved in. As such, proof of principle methods to check the global effect of the miRNA regulation using cellular assays, such as BrdU incorporation for proliferation or nuclear condensation by propidium iodide and caspase 3 activation for apoptosis. The latter was incorporated into a study to induce HL by means of exposure to high frequency noise and aimed to assess the amount of apoptotic hair cells (Patel et al., 2013). From these experiments they learned that the amount of nuclear condensation, an explicit sign of apoptosis, probed with the DNA intercalating agent propidium iodide is comparable following noise exposure. In a different study, set to investigate whether certain miRNAs can promote proliferation of cells in the chick inner ear, basilar papilla were cultured in the presence of BrdU a cell cycle marker that is incorporated instead of thymidine during DNA synthesis (Frucht et al., 2010). Cells were transfected with pre-miRNA181a or anti-miR181a and imaged. A significant number of new hair cells could be observed, providing a role of miR-181a in the pro-proliferative process.

Possibly the most direct method to study the involvement of miRNAs in inner ear mechanisms is in a model animal. Both zebrafish and mice are used to generate knock-out model systems of a single miRNA or miRNA family. Studying these models provides a global indication of phenotypes and can provide information on the targets and the signaling networks in which these miRNAs are involved. More specifically, to study the roles of miR-15a-1 and -18a in zebrafish development, antisense-oligonucleotide MOs were injected into zebrafish 48 h post-fertilization (Friedman et al., 2009). Both morphants showed a reduction in hair cell number and different abnormalities in inner ear structure, indicating that the two miRNAs act in parallel but different pathways. Additional models may be obtained from a resource of miRNA reporter and conditional knockout mouse lines (Park et al., 2012).

## EAR-RELATED PATHOLOGIES AND miRNAs

While not prevalent, a number of mutations in miRNAs have been associated with human HL. The first mutations found were in two unrelated Spanish families (Mencia et al., 2009). This discovery provided strong evidence that two different mutations in the seed region of MIR-96, +13 G>A, and +14 C>A, are sufficient to lead to dysregulation of the miRNA, with the end result of progressive HL. An additional mutation was found in MIR-96 in an Italian family during a screening for miRNA mutations in 882 patients with NSHL (Solda et al., 2012). A mutation in the seed region of miR-96-3p, +57 T>C, is associated with HL in this family with progressive HL. The +57 T>C mutation is predicted to lead to alteration of the secondary structure of the pre-miR-96 hairpin. There was considerable reduction in the expression of both miR-96-5p and -3p. The 5p of a miRNA, together with its complementary strand 3p miRNA, form the pre-miRNA, which is then cleaved by Dicer. While miR-96 transcripts were shown to be reduced in the +13 G < A miR-96 mutation (Mencia et al., 2009), but there was no change in miR-3p, suggesting that the biogenesis of the pre-miRNA is normal. While the mutated miR-96 is degraded, the mechanism is still unknown.

In an effort to determine whether the miRNA-183 cluster is further involved in deafness, predicted target genes of the miR-183 miRNA, expressed in the inner ear, were screened in 150 Americans with autosomal dominant NSHL and 576 Iranians with autosomal recessive NSHL (Hildebrand et al., 2010). A miRNA binding site was predicted in the 3′UTR of radixin, a gene associated with DFNB24 deafness. A variant was found in an Iranian family, c.*95C>A, predicted to alter the binding site of miR-96/182 and create a new miRNA binding site for miR-507 and -557. However, during the validation process, no correlation was found between either of the miRNAs and radixin. It appears that mutations affecting gene regulation of the miR-183 family are not typical causes of a deafness phenotype.

## miRNAs IN AGE-RELATED HEARING LOSS

While hearing impairment does not spare any population, the aging population is hardest hit with this sensory loss. In the aging population, 43% of individuals over the age of 65–75 have a HL (National Academy on an Aging Society)^3^. ARHL has both genetic and environmental contributions. There is growing evidence that miRNAs are involved in cell senescence, death and aging (Inukai and Slack, 2013). To investigate whether miRNAs are involved in regulation of ARHL and the processes leading to it, sensory epithelia were dissected from two mouse strains at several ages, ranging from 21 days after birth (P21) to 16 months (Zhang et al., 2013). They hybridized the isolated RNA from each group on a GeneChip microarray, probing for all known miRNA genes, and differential expression of miRNAs was examined. In both strains, more miRNAs were downregulated from P21 to 9 or 16 month. Moreover, there were a few miRNAs that were differentially expressed in each one of the strains. The data verified that two miRNAs, miR-29a and -34a, which have been implicated in apoptotic pathways, are up-regulated and the two miRNAs, miR-181 and -183, which have been shown to have roles in proliferation and differentiation, are down-regulated

While it is believed that a major cause of ARHL is the death of hair cells, other age-related changes in the central auditory pathways cannot be ruled out. It would therefore be useful to examine the miRNA expression profile in the SOC of aged mice as well. In addition, with the aid of RNA-Seq techniques that have become relatively common and less expensive, it is anticipated that additional miRNAs will be found to play a role in ARHL using this technology.

## miRNAs IN THE MIDDLE EAR

Otitis media (OM) is the most common cause of HL in children. OM is an inflammatory disease of the middle ear mucosa (Lieberthal et al., 2013). While OM is predicted to be multifactorial, with bacterial infections as a contributing factor, its etiology is largely unknown. The cell wall of gram-negative bacteria is partly composed of lipopolysaccharides (LPS), which upon interaction with the host, induce inflammation. Human middle ear epithelial cells (HMEECs), treated with LPS to trigger inflammation, were used to study miRNAs that are differentially expressed in this model system of OM (Song et al., 2011). A gene expression analysis using microarrays led to the identification of 15 differentially expressed miRNAs in HMEECs treated with LPS versus controls, five of which were upregulated and 10 were downregulated. mRNAs that are predicted to be targeted by the upregulated miRNAs are involved in developmental processes, response to biotic stimuli, acute inflammatory responses, and regulation of cell growth, while the downregulated miRNAs are involved in developmental processes, cell differentiation, endocytosis, cell communication, the NFkB cascade, complement activation, innate immune response and cell adhesion. This is the first study to implicate miRNA regulation in OM.

## miRNAs AND APOPTOSIS IN THE INNER EAR

Reactive oxygen species (ROS) are important intercellular messengers; however, when in excess, these species underlie processes such as cell death and apoptosis by modulating the expression of many genes (Circu and Aw, 2010). ROS have shown to be involved in HL and specifically hair cell death (Kopke et al., 1999). Moreover, they have been found in human inner ear perilymph derived from patients with sensorineural HL (Ciorba et al., 2010). To explore whether miRNAs are involved in ROS production in the ear, an *in vitro* cellular model system was used. Tert-butyl hydroperoxide (t-BHP) was used to promote generation of ROS in HEI-OC1 cells derived from the organ of Corti (Wang et al., 2010b). The miRNA expression profile was determined for the t-BHP treated cells; 35 miRNAs were found to be upregulated, while 40 miRNAs were downregulated. The treatment also modulated the expression of many mRNAs, and most relevant, changes in miRNAs were associated with changes in mRNA expression of their predicted targets. Specific examples of predicted miRNA-target pairs were IGF-1, PIK3R1, and PTPN11, which were downregulated, with upregulation of miR-29a, miR-17, and miR-200c, respectively. These results suggest that as a result of oxidative stress, the IGF-1 mediated signaling was altered due to increased transcription of miRNAs in this ROS model.

Antibiotic-induced HL is a major factor in ototoxicity. The potential link between aminoglycoside toxicity and miRNA regulation and its effect on the inner ear was examined (Yu et al., 2010). Kanamycin ototoxicity was induced in mice by subcutaneous injection and inner ears were analyzed. In response to the treatment, the mice exhibited a reduced ABR response, which deteriorated as a function of time. Cell death, evaluated by the TUNEL assay, was increased in particular in the stria vascularis, supporting cells and spiral ganglion cells. Due to their previously known role in apoptosis, levels of the miR-34 family were examined in RNA extracted from cochleae of treated mice by qRT-PCR analysis. Both miR-34a and miR-34c were significantly elevated, as compared to untreated controls. This data suggested that apoptosis in the inner ear, followed by hearing damage in this model previously linked to programmed cell death, is partly mediated by members of the miR-34 family.

## miRNAs AND REGENERATION IN THE INNER EAR

An early study in miRNAs and regeneration appeared soon after the first report of miRNAs in the mammalian inner ear (Tsonis et al., 2007). The adult newt has the ability to regenerate body parts, including the cells of the inner ear, by transdifferentiation of terminally differentiate cells. In an effort to identify potential changes in gene expression during this process, miRNA profiles were examined during hair cell (and eye lens) regeneration. The level of expression of let-7 miRNAs were found to be significantly reduced. While there have been several studies on lens regeneration and miRNAs since then, no additional studies on the ear have been reported.

The avian auditory sensory epithelium, the basilar papilla, is different from the mammalian sensory epithelium not only in its structural organization, but also in its ability to regenerate following hair cell loss. As in the mammalian cochlea, in the basilar papilla, both hair cells and supporting cells can be found. Upon injury of any kind, such as noise or ototoxicity, there are new hair cells produced from de-differentiation of supporting cells (Balak et al., 1990). Supporting cells of birds that were exposed to acute noise will re-enter cell cycle and within 4–5 days of trauma, new hair cells could be found in the basilar papilla (Stone and Cotanche, 2007).

To elucidate the role of miRNAs in the intracellular signaling pathways of chick hair cell regeneration, forskolin, a compound known to induce proliferation of supporting cells to hair cells, was applied on basilar papilla cultures (Frucht et al., 2010). The miRNA expression profile was evaluated using microarray analysis. miR-181a, which was greatly enriched in the proliferating basilar papilla and as it had previously been identified to have a role in promoting proliferation in a human leukemia cell line, was selected as a hair cell proliferation candidate. Overexpression of miRNA-181a was indeed able to stimulate proliferation within the basilar papilla, with new cells labeling with the hair cell marker myosin VI. A subsequent study further explored miR-181a involvement in the pro-proliferative processes in chickens (Frucht et al., 2011). To this end, the hair cells of the basilar papilla were destroyed using streptomycin. Down-regulation of this miRNA inhibited proliferation during regeneration, rather than preventing hair cell death, providing not only its mechanism in the process, but a promising candidate for regeneration.

## FUTURE OF miRNAs IN THE INNER EAR

High-throughput sequencing for RNA, dubbed RNA-seq, has facilitated the study of miRNAs dramatically (Oshlack et al., 2010). RNA-seq is being used to evaluate miRNA expression with a comparison of multiple sets of conditions. The large datasets obtained can be narrowed down to a smaller set of miRNAs to be evaluated in their role in regulation and gene expression. While RNA-seq has been used in multiple fields to identify and characterize miRNAs, this technology has still not been exploited in the inner ear field.

The field on ncRNAs in the mammalian inner ear is still very much in development. While there has been tremendous progress in the last decade, there are areas that are still in their infancy. One such area is that of lincRNAs. lincRNAs are relatively long stretches of RNA larger than 200nt (Ponting et al., 2009). They were identified relying on knowledge from protein-coding transcripts. Both coding and non-coding transcripts have particular chromatin signatures consisting of H3K4me3 and H3K36me3. By identifying K4–K36 domains that lay outside known protein-coding loci, lincRNAs could be methodically identified (Guttman et al., 2009). Unlike miRNAs, lincRNAs have no shared structural characteristics; their biogenesis and processing is unique, as well as their mode of action. Therefore it is not straightforward to identify and study them. An additional factor that hampers the research in the field of these new species is that lincRNAs are extremely cell- and tissue-specific, making their discovery particular to each system. Although there is some evolutionary conservation between species, it is much less prominent than the one observed in coding RNA transcripts, adding to the complexity of their identification. As opposed to miRNAs, the biological functions of lincRNAs are largely unknown. Moreover, some of the already described roles of the lincRNA are variable and are not necessarily mutually exclusive (Da Sacco et al., 2012). lincRNAs have been found to act as gene activators, gene suppressors, cis and trans gene expression regulators, and chromatin modificators.

LincRNAs have been shown to play a critical role in the development and regulation of the sensory systems. As for the long ncRNA species, lincRNAs have been found expressed in the mouse retina (Mustafi et al., 2013) and suggested to be associated with retinal and visual maintenance in mammals. Another study showed that the taurine upregulated gene 1 (Tug1) lincRNA is required for differentiation of the murine retina acting via regulation of the cell cycle (Young et al., 2005). Two studies have reported lincRNAs in the inner ear. MEG3, a lincRNA implicated in leukemia (Heuston et al., 2011), was examined following microarray detection experiment of enriched transcripts in a mouse inner ear library (Manji et al., 2006). Following detection of its expression in the developing otocyst, spiral ganglion, stria vascularis, Reissner’s membrane, and greater epithelial ridge (GER), as well as hair and supporting cells, MEG3 was hypothesized to play a role in pattern specification and differentiation during otocyst development and maintenance terminally differentiated cochlear cells. *Rubie* was identified as an inner-ear specific lincRNA upstream of the *Bmp4* gene (Roberts et al., 2012). Rubie was predicted to be the gene mutated in epistatic circler (*Ecl*) mice, contributing to its vestibular phenotype. There is clearly room for a comprehensive investigation of lincRNAs in the inner ear.

One of the most exciting developments in the field has been the generation of a novel *in vitro* model system for the inner ear (Koehler et al., 2013). Mouse embryonic stem cells (ESC) were differentiated in a step-wise manner into a 3D culture of vestibular sensory epithelia. These cells showed characteristics of innate hair cells; they were able to take up FM1-43 dye and exhibited voltage-dependent currents. Moreover, ribbon synapses were formed between the hair cells and neighboring neurons in the 3D culture. These cells may serve as a substrate for investigating additional aspects of RNA regulation and lead to identification of additional RNA species in the inner ear.

## CONCLUSION

miRNAs are being developed as therapeutics for breast cancer (Piva et al., 2013), rheumatic diseases (Pers and Jorgensen, 2013), and hepatitis C virus infection (Lindow and Kauppinen, 2012; Janssen et al., 2013), which are already involved in Phase 2 clinical trials. The major limitations in miRNA research in the inner ear are the lack of robust cell lines and the inability to gain access to human tissue in an efficient manner. Nevertheless, the identification of hundreds of miRNAs in the auditory system and the elucidation of the function of many of these miRNAs and their targets holds promise for their use in therapeutics one day. A deeper understanding of the regulatory elements involved in the diseased state of the ear, including hearing impairment, cholesteatoma, OM, and vestibular schwannomas can be reached, with miRNAs serving as a potential source of regeneration therapies and relevant pharmaceutical studies. The ability to generate stem cells may open up further avenues for RNA regulation studies.

## Conflict of Interest Statement

The authors declare that the research was conducted in the absence of any commercial or financial relationships that could be construed as a potential conflict of interest.

## Footnotes

1 http://www.nidcd.nih.gov/health/statistics/hearing.html

2 http://www.mirbase.org

3 http://www.agingsociety.org/agingsociety/
